# The complete chloroplast genome sequence of *Gynura cusimbua* (D. Don) S. Moore

**DOI:** 10.1080/23802359.2021.1997104

**Published:** 2021-12-22

**Authors:** Lei Mei, Yueyi Zhu, Wei Fu, Yu Kang, Hongqing Yin, Hao Chen

**Affiliations:** a Enshi Tujia & Miao Autonomous Prefecture Academy of Agricultural Sciences, Enshi, China; b College of Agriculture and Biotechnology, Zhejiang University, Hangzhou, China

**Keywords:** *Gynura cusimbua*, complete chloroplast genome, phylogenetic analysis

## Abstract

*Gynura cusimbua* (D. Don) S. Moore is a favorite food vegetable and traditional folk medicine. The chloroplast genome information, of *G. cusimbua*, was introduced and released in this study. The complete chloroplast genome was characterized as 156, 684 base pairs (bp) in length. The circle gDNA contained four segments, namely LSC (large single copy), SSC (small single copy) and two IRs (inverted repeats), which was 86, 834 bp and 18, 414 bp and 25, 718 bp in length separately. The total GC content was 36.88%. A total of 125 genes were characterized in the chloroplast genome, where 84, 33 and 8 genes were for coding-proteins, tRNA and rRNA respectively. The phylogeny tree demonstrated that *G. cusimbua* was clustered with *Jacobaea valgaris* and *Senecio valgaris*. This study would fill a vacancy of chloroplast genome information involving *G. cusimbua*, and provide new genetic resources for the study on *Senecioninae*.

## Introduction

*Gynura cusimbua* (D. Don) S. Moore 1912 is a favorite food vegetable worldwide. Simultaneously, it was employed as a traditional folk medicine in India and China (Ma et al. 2020). The phytochemical studies on *G. cusimbua* showed the presence of phenylpropanoid glycosides, fat-soluble components and volatiles (Ma et al. 2020). However, there were still known rarely on its genetic information. Here, we released the complete sequences of chloroplast genome, which would offer valuable gene information for further studies.

The seeds of *G. cusimbua* was gifted from Yunnan University, China. Seeds were grew in garden soil and kept in cabinet, as conditions of 26 ± 2 °C, Light/Dark: 16 h/8h, 60% relative humidity, at the Agriculture Station, Zhejiang University (120°5′29″E, 30°17′47″N). Fresh leaves were sampled and subsequently extracted the total DNA via general CTAB method. The specimen, with the accessory number *GC-LM002*, was deposited in the Museum of CAB, Zhejiang University (leimei@zju.edu.cn). The whole-genome sequencing was performed by *Novogene biotechnologies Inc*. (Tianjin, China) with *Illumina Hiseq 2000* platform. A genomic shotgun library with an insertion size of 150 bp was constructed, then the clean data were *de novo* assembled using *NOVOPlasty* (*Version 3.8.1*) (Dierckxsens et al. 2017). Finally, the assembled chloroplast genome was annotated by PGA (Qu et al. 2019) and GeSeq (Tillich et al. 2017).

**Figure 1.**
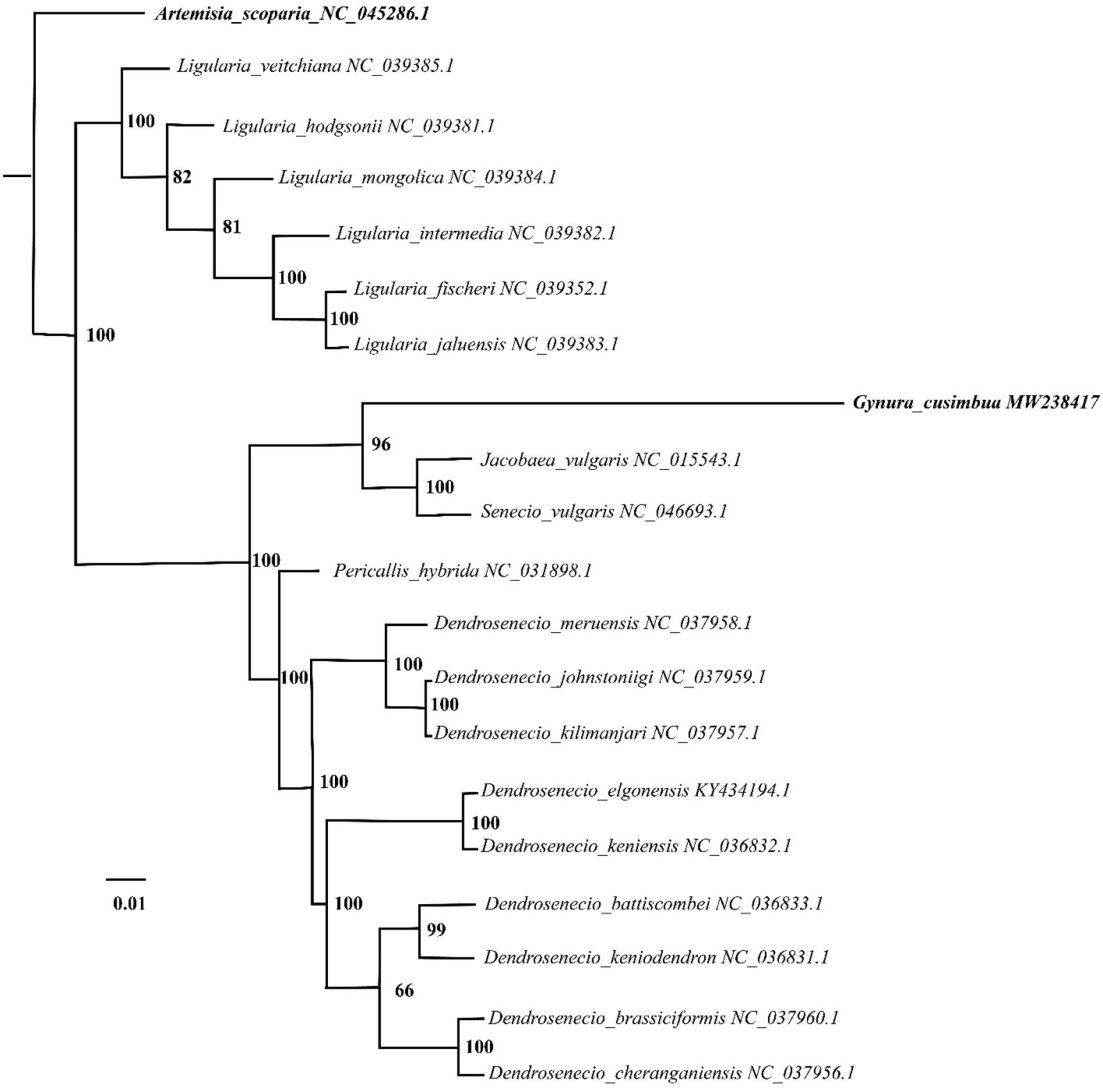
The Maximum-Likelihood (ML) phylogenetic tree of 20 complete chloroplast genomes: Gynura cusimbua is showed with bold italic text for highlight, Artemisia scoparia as an out-group and showed with bold italic text. The numbers adjacent to the nodes denote bootstrap support values from 1000 replicates.

The complete chloroplast genome, of *G. cusimbua*, was characterized as 156, 684 base pairs (bp) in length. The LSC (large single copy) and SSC (small single copy) is 86, 834 bp and 18, 414 bp respectively, which accounts for 55.42% and 11.75% in the whole circle DNA. Additionally, IRa and IRb shared the same length with 25, 718 bp. The percentage of GC content was 36.88. 125 genes were characterized in the chloroplast genome, in which 84, 33 and 8 genes were for coding-proteins, tRNA and rRNA respectively. To analyze the *G. cusimbua* phylogenetic position within *Senecioninae* lineage, 20 chloroplast genomes were employed to align and construct phylogenetic tree by *MAFFT* (*V7.407*) (Katoh and Standley 2013) and *IQtree* (*Vesion 1.7*) (Nguyen et al. 2015), correspondingly. The results indicated that *G. cusimbua* was clustered with *Jacobaea valgaris* and *Senecio valgaris* (Figure 1).

## Data Availability

The genome sequence data that support the findings of this study are openly available in GenBank of NCBI at [https://www.ncbi.nlm.nih.gov] (https://www.ncbi.nlm.nih.gov/) under the accession no. MW238417. The associated BioProject, SRA, and Bio-Sample numbers are PRJNA722038, SRS8704631, and SAMN18743908, respectively.

## References

[CIT0001] Dierckxsens N, Mardulyn P, Smits G. 2017. NOVOPlasty: *de novo* assembly of organelle genomes from whole genome data. Nucleic Acids Res. 45(4):e18. 28204566 10.1093/nar/gkw955 PMC5389512

[CIT0002] Katoh K, Standley DM. 2013. MAFFT multiple sequence alignment software version 7: improvements in performance and usability. Mol Biol Evol. 30(4):772–780. 23329690 10.1093/molbev/mst010 PMC3603318

[CIT0003] Ma Q, Wei R, Zhong G, Sang Z. 2020. Neuroprotective flavonoids from the aerial parts of Gynura cusimbua. Chem Nat Compd. 56(4):725–728.

[CIT0004] Nguyen LT, Schmidt HA, von Haeseler A, Minh BQ. 2015. IQ-TREE: a fast and effective stochastic algorithm for estimating maximum-likelihood phylogenies. Mol Biol Evol. 32(1):268–274. 25371430 10.1093/molbev/msu300 PMC4271533

[CIT0005] Qu XJ, Moore MJ, Li DZ, Yi TS. 2019. PGA: a software package for rapid, accurate, and flexible batch annotation of plastomes. Plant Methods. 15:50. 31139240 10.1186/s13007-019-0435-7 PMC6528300

[CIT0006] Tillich M, Lehwark P, Pellizzer T, Ulbricht-Jones ES, Fischer A, Bock R, Greiner S. 2017. GeSeq – versatile and accurate annotation of organelle genomes. Nucleic Acids Res. 45(W1):W6–W11. 28486635 10.1093/nar/gkx391 PMC5570176

